# Food for Thought: Proteomics for Meat Safety

**DOI:** 10.3390/life13020255

**Published:** 2023-01-17

**Authors:** Svetlana Tarbeeva, Anna Kozlova, Elizaveta Sarygina, Olga Kiseleva, Elena Ponomarenko, Ekaterina Ilgisonis

**Affiliations:** Institute of Biomedical Chemistry, 119121 Moscow, Russia

**Keywords:** meat packing plant, meat processing, bacteria, food safety, *Salmonella*, *Listeria*, *Pseudomonas*, antibiotics, resistance

## Abstract

Foodborne bacteria interconnect food and human health. Despite significant progress in food safety regulation, bacterial contamination is still a serious public health concern and the reason for significant commercial losses. The screening of the microbiome in meals is one of the main aspects of food production safety influencing the health of the end-consumers. Our research provides an overview of proteomics findings in the field of food safety made over the last decade. It was believed that proteomics offered an accurate snapshot of the complex networks of the major biological machines called proteins. The proteomic methods for the detection of pathogens were armed with bioinformatics algorithms, allowing us to map the data onto the genome and transcriptome. The mechanisms of the interaction between bacteria and their environment were elucidated with unprecedented sensitivity, specificity, and depth. Using our web-based tool ScanBious for automated publication analysis, we analyzed over 48,000 scientific articles on antibiotic and disinfectant resistance and highlighted the benefits of proteomics for the food safety field. The most promising approach to studying safety in food production is the combination of classical genomic and metagenomic approaches and the advantages provided by proteomic methods with the use of panoramic and targeted mass spectrometry.

## 1. Introduction

The safety of meat products is one of the most important current and future challenges requiring the attention of the food industry, scientists, and public health authorities.

Despite the significant advancement of sequencing technologies and the giant pools of NGS data accumulated, robust, cost-effective, and reliable techniques, allowing the annotation of the microbial status of food, remain a challenge for the scientific community. Today, MS-based technologies to detect foodborne pathogens complement the classical methods and NGS. We would like to highlight two directions in the trends in pathogen proteomics. The first direction, which has obvious practical value, is supported by the efforts of the entire proteomic community to ensure in-process control and reduce batch-to-batch variations, especially in label-free techniques.

Taking into account the short duration of the “life” of certain meat products, there is a need for robust and cost-effective methods for pathogen detection, applicable both in the laboratory and in marketable products [1]. We expect the classic “from farm to fork” path to become “from farm through mass spec to fork” soon, especially if the community develops reliable identification criteria, such as standardized lists of proteotypic peptides that could be used as reliable proof of protein presence.

The second hot topic in the field of foodborne pathogens is of high fundamental importance in the context of host–pathogen interactions covering all stages of infection from invasion to dissemination. It would not be an exaggeration to say that the interactions between molecules enable life. To understand the molecular mechanisms behind the life cycle of common foodborne infections, a complete map of the interactions between the proteins and metabolites of pathogens, food products, and, in fact, the end consumer must be formed. We are seeing similar interactive initiatives in the fight against seed-borne diseases in wheat, which lead to the reduced quality and quantity of the crop, and expect similar initiatives from the research community focused on meat products [2,3].

To date, genomic and transcriptomic methods have a clear advantage over proteomic methods in the field of food production state monitoring due to several reasons. Genomic and transcriptomic methods are more mature and have a longer history of use, the instruments are cheaper and, accordingly, more common. If we compare the genomic and proteomic research intensities for food production safety monitoring, there are 6-times more publications that correspond to genomic research (3122 articles according to PubMed, December 2022) than to proteomic research (500 articles according to PubMed, December 2022). In addition, some mechanisms of bacterial resistance are based on genome changes. Furthermore, for each technology, we can observe a gap between the fundamental research and the applied application in food production safety monitoring for both genomics and proteomics. The turning point is, among other things, the emergence of regulatory documents that allow the use of technology to assess the safety of production. WGS (whole-genome sequencing) gained acceptance as a prospective surveillance tool for foodborne illness in 2016 [4,5,6], and now it is increasingly replacing traditional microbial typing and characterization techniques, providing faster and more precise answers [7]. As for proteomics, there are no regulatory documents for its use for food safety monitoring; the normative and methodical bases are still under construction [8].

At the same time, proteomic methods give an opportunity to detect rapid changes in the molecular responses of bacterial cells to aggressive stimuli such as antibiotic damage. Therefore, the usefulness of these methods will provide more information about the mechanisms of bacterial resistance and the strains’ life cycles in food production.

Traditionally, meat spoilage pathogens are identified with culture-based methods focused on the biochemical, physiological, and morphological properties of the microorganisms under study. These classical methods typically require several days to obtain the results. Moreover, these methods do not provide valuable information on the dead pathogen cells or toxins. Despite the tremendous democratization of NGS methods, high-throughput mass spectrometry-based proteomics has been recognized as an essential tool to reliably identify and quantify thousands of proteins in complex biological matrices.

Foodomics renders mass spectrometry as a major “working horse” for its “direct” nature of qualitative and quantitative data on the molecules of interest with great performance and adaptability.

Technical progress and scientific curiosity are bringing to the market new MS devices with increased robustness, sensitivity, and selectivity, and decreased sample demand. The variety of mass spectrometric methods is expansive; however, in a broad sense, all MS approaches can be divided into panoramic (or “shotgun”) and targeted groups [9,10]. In the context of food safety, the panoramic MS is used for the exploration of a meat proteome from a bird’s eye view to massively identify potential biomarkers of foodborne pathogens. Several commonly used variations of the panoramic strategy are focused on the intact proteins (“top-down”) and individual peptides (“bottom-up”) in which selective proteases digest the proteins into proteotypic peptides that are submitted into the MS system [11]. In turn, the methods of targeted proteomics are tailored to quantify the preselected peptides with high sensitivity and precision.

Obviously, all fundamental and practical questions cannot be incorporated into one analytical device, providing panoramic or targeted snapshots of the sample under study. The range of problems that proteomics solves is wider than just the panoramic or targeted search. Often, a researcher analyzes proteins with specific biochemical properties (charge, size, or hydrophobicity), conformational changes, post-translational modifications, or certain interacting partners [12,13]. To answer all these questions, researchers use panoramic or targeted mass spectrometry but enhance it with a whole arsenal of superior supporting technologies: e.g., sample preparation (antibody depletion or enrichment), fractionation (two-dimensional gel electrophoresis or multidimensional chromatography), and quantification (iTRAQ or label-free) [14,15]. Thus, speaking of panoramic or targeted mass spectrometry, we describe an extensive range of methods that can comprehensively explore a particular sample’s proteome. The current review summarizes the significant achievements in proteomic analysis for food production. We summarized the most up-to-date proteomic achievements to highlight the relevance of this omics discipline in elucidating bacterial lifestyle, developing food safety biomarkers, and innovating food protection strategies.

The first part of the article is devoted to the methods used for microbial strain analysis, focused on food pathogens. Further review is focused on three key strains (*Salmonella enterica*, *Listeria monocytogenes*, and *Pseudomonas aeruginosa*) and the poisoning products that cause infectious diseases. The final part includes data on the markers of disinfectant and antibiotic resistance.

It is important to note that we have chosen the PubMed database as a source of publications on the bacterial resistance specific to food production. Despite the fact that this database is not specialized for the problems of food production, it has a number of advantages in the context of our study. First of all, it is one of the most complete and structured databases in the field of biological research and very well adapted for the automatic analysis of a large number of publications. Secondly, omics technologies are at the crossroads of many natural sciences, including biology, medicine, and agriculture, and the peculiarities of their use in the context of bacterial research can be published in various thematic journals. At the same time, proteomic methods are still quite expensive and highly specialized; therefore, they are more often used in fundamental research than in commercial food production control.

## 2. Materials and Methods

The research was performed using a combination of automatic text-analysis algorithms, ScanBious [16,17] and biocuration [18]. The automatic text-analysis algorithms used in this work have previously shown their effectiveness for reconstructing metabolic pathways [19], assessing the current state of the selected subject area [20], and investigating trends in scientific research in the field of biomedicine [21]. The PubMed/MEDLINE library of scientific publications in biology and medicine was used as a data source [22]. Each article was associated with a set of keywords (MeSH (Medical Subject Heading) terms) assigned by the curators of the resource for indexing and briefly characterizing the content of each publication. ScanBious is a free-ware Web-system for highlighting key concepts revealed from PubMed abstracts and related MeSH terms; it provides an interface to the transparent algorithm, which relies on the co-occurrence of two terms in one abstract [20]. One can make a search query to ScanBious and get a list of relevant articles similar to the PubMed/MEDLINE system. Unlike PubMed/MEDLINE, the user can process the obtained set of publications: select the most relevant to the search query articles and their abstracts, extract their MeSH terms, select target MeSH terms, and visualize them as a semantic network based on the measure of interrelation (Jaccard index). The nodes of the semantic network are the objects (MeSH terms) selected by the user and the links of which are a list of publications in which both objects are described [20]. The size of the nodes reflects the number of publications; the thickness and the color of the edge–Jaccard’s index.

Using ScanBious, a semantic map of the subject area was built. Visualization was carried out using the frequency characteristics of the MeSH terms of the publications corresponding to the search query [“meat production” OR “meat products” OR “meat processing” OR “meat industry” OR “meat processing products”, “meat” OR “food production” OR “food industry”].

About 48,000 relevant publications were processed. The nodes of the map are the MeSH terms, and the edges between the nodes reflect the joint mention of the terms in the same study. The ScanBious system provides an opportunity to work with the publications associated with the nodes and the edges of the semantic map. Thus, after a short dive into the subject area using the semantic map, detailed information can be obtained from the texts of the relevant articles.

The hierarchical structure of the organization of the MeSH dictionary of terms also allows for the limiting of the display, on the semantic map of the subject area, of terms related to specific categories. Thus, one can set the visualization of the MeSH terms related to the subject area and characterize the methods of study (MeSH category “Analytical, Diagnostic and Therapeutic Techniques, and Equipment”) or, for example, diseases (“Diseases”).

## 3. Results and Discussion

### 3.1. Subject Area Semantic Network Description

**Figure 1:**
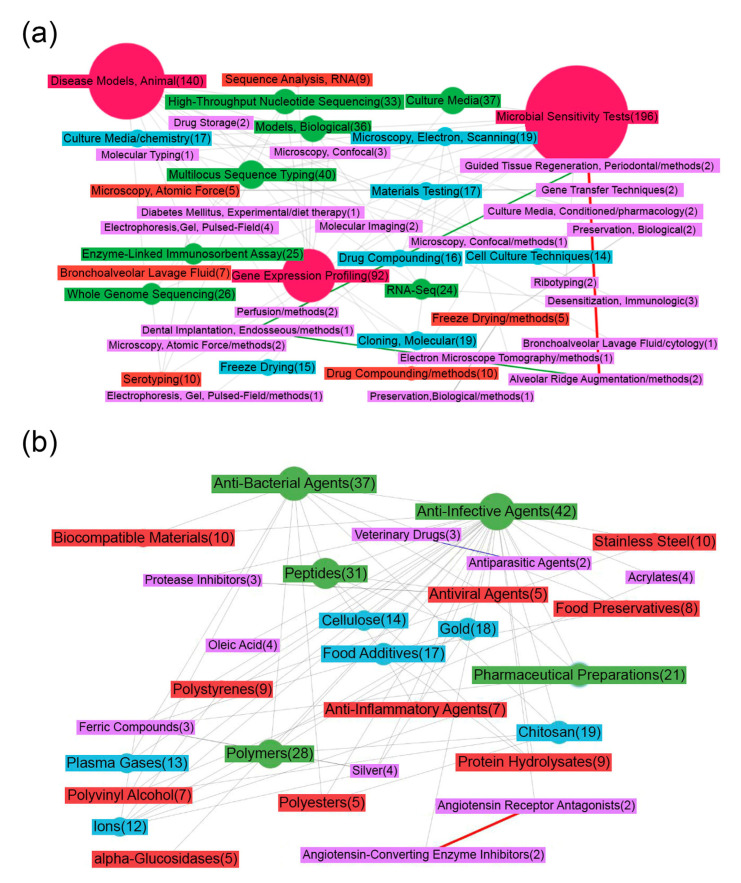
Map of MeSH terms resulting from ScanBious processing of publications in the PubMed/MEDLINE system associated with food (meat) production research. The map displays the MeSH terms related to the category “Analytical, Diagnostic and Therapeutic Techniques, and Equipment” (**a**) and “Chemical and Drug” (**b**). The number in parentheses indicates the number of articles in which the term was found. The color and thickness of the connecting lines and nodes reflects the degree of interaction between the two terms: the thicker the line, the more articles included both terms simultaneously. The gray color corresponds to Jaccard index indices with values of 0.2 and below, the blue color corresponds to values from 0.2–0.4, the green corresponds to values from 0.4–0.6, the yellow corresponds to values from 0.6–0.8, and the red corresponds to values of 0.8 and above.

Figure 1a shows a map of the MeSH terms that characterize the publications downloaded on demand (see the Materials and Methods section). It draws attention to the fact that the most researched issue related to food production is the creation of microbiological control tests (“Microbial Tests”). To profile and assess the composition of microbial communities, genomic and transcriptome sequencing is mainly used; the semantic map contains nodes that confirm this statement (for example, “High-Throughput Nucleotide Sequencing”, “RNA sequence Analysis”, “Multilocus Sequence Typing”, “Gene Expression Profiling”, “Whole genome sequencing”) and various microscopy methods (electron-scanning, atomic force, confocal). Proteomic approaches for the analysis of microbial communities (“Gel Electrophoresis”, “Enzyme-Linked Immunosorbent Assay”) are much less widely used.

An essential task in determining the composition of microbial communities is assessing resistance to various types of disinfectants, for example, antibiotics; the effectiveness of measures aimed at the safety of food production directly depends on this. The scientific works in this area characterize the MeSH terms associated with drugs (“Drug Compounding”, “Drug Storage”) and methods for studying antibiotic resistance in animals and cell lines (“Models Biological”, “Cell Culture Techniques”, “Disease Models, Animal”). Figure 1b depicts the MeSH terms from the category “Chemical and Drug”. Most of the publications were indexed using high-level MeSH terms (“Anti-Infective Agents”, “Protease Inhibitors”), not defining exact compounds.

In a study [23] on the metagenetic analysis of food production, it was shown that the leaders among infectious agents are *Proteobacteria* (found in 71.37% of bacterial communities) and *Actinobacteria* (79.72%). As biofilms mature, the species composition changes somewhat, but *Pseudomonas*, *Enterobacteriaceae*, and *Firmicutes* are also widely encountered [24].

*Pseudomonas* are ubiquitous bacteria due to their versatile set of proteins involved in metabolism [25]. Studies conducted over several decades on different continents have shown that *Pseudomonas* from raw materials or treated surfaces, cooling air, or water can proliferate in a food product, especially under aerobic conditions [26]. At the end of the shelf life, *Pseudomonas* dominates and spoils the meat through proteolytic, lipolytic, saccharolytic, and biosurfactant processes [27]. *Pseudomonas aeruginosa* remains one of the most important species in terms of antibiotic resistance due to its high tendency to acquire polyresistance [28,29,30].

Gram-negative *Salmonella enterica* (family *Enterobacteriaceae*) is an intracellular parasite that invades small intestinal mucosal cells. Entering the human body with food, this bacterium causes the dangerous disease salmonellosis, which accounts for 25% of all human diarrheal diseases, ranging from mild infections to death [31]. *Salmonella* strains with resistance to multiple antimicrobials have long spread in developed and developing countries [32]. It is generally accepted that, for the most part, such strains are of zoonotic origin. They acquire resistance due to the use of antimicrobials for prophylactic or therapeutic purposes in the food industries.

*Listeria monocytogenes* is a member of *Firmicutes*, a rod-shaped, Gram-positive pathogenic bacterium that is a facultative intracellular parasite. Once in the human body, *Listeria monocytogenes* causes the dangerous invasive disease listeriosis, an infection that accounts for about 28% of deaths due to foodborne diseases [33]. This pathogen can cross three critical barriers in the human host, namely the intestinal epithelium, the blood–brain barrier, and the placenta, and can subsequently spread to other organs. *Listeria monocytogenes* can grow at refrigeration temperatures [34], thereby increasing the potential hazard of eating refrigerated and ready-to-eat (RTE) foods.

Bacterial resistance research is complicated due to the bacteria’s ability to form biofilms. A biofilm is a structured community of bacterial cells enclosed in a self-produced matrix that adheres to inert or living surfaces, including tissues, industrial surfaces, and artificial devices, such as intrauterine contraceptive devices, implants and prosthetic medical devices, catheters, dental materials, cardiac valves, and contact lenses. Biofilms form when bacterial colonizers adhere to surfaces in aqueous environments and excrete a slimy, glue-like substance composed of exopolysaccharides (EPS) [35]. It gives them consistency and resistance to antibiotics and disinfectants [36,37]. Biofilm formation and proliferation affects many aspects of public health and industrial processes, including being a source of contamination in the food and beverage industries [38,39,40].

The essence of this problem is that bacteria, organized on any surface as complex communities (biofilms), acquire qualitatively new properties. The biofilm bacteria can increase resistance to immune system effectors, antibiotics, and disinfectants. Food preservatives, disinfectants, and resistance to antiseptics are under-explored [41], and biofilms complicate the research process.

**Figure 2:**
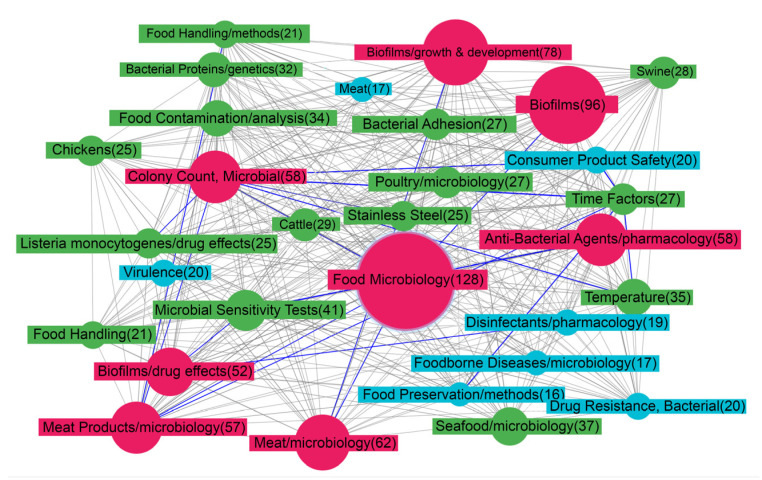
Map of MeSH terms resulting from ScanBious processing of publications in the PubMed/MEDLINE system associated with food (meat) production research. The map displays the MeSH terms related to the category “Phenomena and Processes”. The color and thickness of the connecting lines and nodes reflects the degree of interaction between the two terms: the thicker the line, the more articles included both terms simultaneously. The gray color corresponds to Jaccard index indices with values of 0.2 and below, the blue color corresponds to values from 0.2–0.4, the green corresponds to values from 0.4–0.6, the yellow corresponds to values from 0.6–0.8, and the red corresponds to values of 0.8 and above.

One of the leading positions in Figure 2 belongs to the term “Biofilms”. The closest nodes depict some conditions that affect biofilm development: “Temperature”, “Time-factors”, “Bacterial adhesion”. A given species or strain of bacteria responds to environmental conditions via a finite number of key regulatory factors and pathways, which affect the enzymatic and structural elements that are needed for biofilm formation and dispersal. Among the conditions that affect biofilm development are temperature, pH, O2 levels, hydrodynamics, osmolality, the presence of specific ions, nutrients, and factors derived from the biotic environment. The integration of these influences ultimately determines the pattern of behavior of a given bacterium with respect to biofilm development [42].

### 3.2. Genetic Determinants of Resistance

Bacteria, including the considered *Salmonella enterica*, *Listeria monocytogenes*, and *Pseudomonas aeruginosa*, acquire resistance to antimicrobial agents through the following mechanisms: (1) structure change of the antibiotic target (mutation), (2) enzymatic degradation or restructuring of the antibiotic, (3) active cell release of antibiotics or efflux, (4) reduced permeability to antibiotics, (5) use of alternative metabolic pathways, (6) and formation of biofilms [43,44].

Resistance mechanisms may be driven by acquiring three types of mobile genetic elements: self-transferring plasmids, mobilizable plasmids, and conjugative transposons [45]. Regarding antibiotic and disinfectant resistance in bacteria, researchers often consider genotypic research methods. Isolated bacterial strains from patient material or food are subjected to antibiotic susceptibility tests. The bacterial strains are treated with the antibiotic of interest after which the colony’s inhibition zone’s size is determined. Then, the researcher’s task is reduced to a search for the resistance gene and the donor plasmid.

Determining the resistance genes and assessing their expression at the transcriptomic level do not recreate the complete picture of the systemic rearrangements of cellular processes under the stress caused by antibiotics. Currently, studies are underway to establish the position of small non-coding RNAs in rearranging metabolic networks under external stress, including that caused by antibiotics [46,47]. RNA-binding proteins play an essential role in modulating the interactions of regulatory small non-coding RNAs [48]. In addition to the metabolic rearrangements in response to stress in bacteria, proteins can undergo post-translational modifications, which can be involved in developing resistance [49].

Combining several omics technologies makes it possible to recreate the complete network of molecular interactions. Proteomics is evolving rapidly, and its flexibility and potential have yet to be fully exploited. Sidoli et al. even supposed that, in the next ten years, every biological institute will have at least one mass spectrometer [50]. Mass spectrometry (MS) provides valuable insights into the signaling, post-translational changes, and microbial–host interactions relevant to bacterial pathogenesis. Although transcriptomics still outperforms mass spectrometry in several ways (for example, in terms of the estimated cost of the experiment), proteomic methods provide many undeniable advantages.

The results of the literature analysis devoted mainly to the genotypic methods for studying the resistance in selected strains are presented in Table 1. This table accumulates information on the available resistance determinants: the gene name, the corresponding UniProt ID, and the neutralizable antimicrobial agent.

According to Table 1, most of the protein products of resistance genes have the “unreviewed” status according to the UniProt database. This means that computational methods predicted the sequences of these proteins. These facts define proteomics as the missing link in the chain of research to establish resistance mechanisms and search for alternative targets for treating bacterial infections.

### 3.3. Proteomic Methods for Studying the Microbiome of Food Industries

(a) Panoramic mass spectrometry

Panoramic mass spectrometry, or shotgun proteomics, determines the complete set of proteins in a complex multi-component cell extract. Shotgun proteomics uses a liquid chromatograph with a tandem mass spectrometer. This method is considered panoramic because the mass spectrometer registers all peptide ions of the isolated proteins. Shotgun proteomics for studying the mechanisms of the development of resistance in bacteria can be applied similarly to the genotypic approach to measure protein concentrations in strains pretreated with antimicrobial agents, identify overexpression, and try to establish a relationship.

Coldham et al. [54] studied the physiological response of *Salmonella enterica* (*Typhimurium* serovar) to fluoroquinolone antibiotics using proteomic methods. The protein expression was assessed using two-dimensional HPLC-MS. The increased expression of AcrAB/TolC proteins (efflux pump proteins) is associated with the development of antibiotic resistance. Qi et al. also associated the AcrB efflux pump protein with antibiotic resistance, but they also mentioned the BaeSR protein system [57]. They suggested that these proteins play a role in the phosphorylation of other *Salmonella enterica* proteins, which, in turn, leads to the development of drug resistance.

Ultra-high-performance liquid chromatography with tandem mass spectrometry is often used to detect bacterial aggression factors. For example, the authors of the following work identified enterotoxins of *Staphylococcus aureus*, which has 15 variants of the toxin [79].

Similarly, using tandem mass spectrometry with electrospray ionization coupled with liquid chromatography (LC–ESI–MS/MS) in a large-scale study [68], 395 proteins with a direct role in the pathogenicity of Listeria were identified. These proteins were virulence factors, toxins, antitoxins, and molecules associated with antibiotic resistance or resistance to toxic substances. In particular, β-lactamases, MarR transcription regulators involved in the transduction of β-lactamase metallopeptidase M56, penicillin-binding proteins, etc., were found among the latter.

As mentioned earlier, the post-translational modifications of proteins also impact bacterial resistance to antibiotics [55]. For example, Li et al. proved that the acetylation status of lysine in the proteins associated with fluoroquinolone resistance varies in resistant and susceptible strains of *Salmonella enterica*. A high content of acetylated lysine characterizes the proteins of resistant *Salmonella enterica*.

Similarly, in work [80] devoted to the resistance of *Pseudomonas aeruginosa* to ofloxacin, post-translational modifications of PheA and SpuC proteins (succinylation and acetylation) were revealed. The study showed that the modifications of pheA and spuC modulate resistance to ofloxacin. The presence of post-translational modifications on these two proteins could direct the protein to a different cellular function and promote the survival of *P. aeruginosa*.

The changes in the metabolic pathways that cause the development of antibiotic resistance can also be captured using proteomic methods. Erdmann J. and colleagues established the quantitative ratio of the core proteome for 27 clinical isolate samples using the DIA technology (data independent acquisition) on the nanoLC-ESI-MS/MS platform (TripleTOF 5600+) [81]. Genomic and transcriptomic analyses were also performed for each sample. The study of soft-core biofilm proteins showed that, despite significant variability between the strains, there was an increase in the concentration of proteins involved in the iron sequestration and iron metabolism processes (PchDG, FptA), *Pseudomonas* quinolone signal transduction (PQS) (PqsBCDH), phenazine biosynthesis (PhzB1, PhzB2, PhzE2, PhzF2, PhzM), outer membrane proteins (OprCFG, MexI), and fatty acid biosynthesis (FabG).

Panoramic proteomics can be successfully incorporated into multi-omics projects to visualize the multi-dimensional nature of foodborne pathogens in the context of genomics, proteomics, metabolomics, and clinical data. The major advantage of panoramic MS over targeted techniques is that it fuels non-hypothesis-driven proteomic research, making it superior for the early stages of biomarker discovery when dealing with complex biological matrices. Another MS benefit is the ability to explore the processes performed by the whole microbial community at the time of sample collection in time- and cost-effective ways.

(b) Targeted mass spectrometry

In the targeted proteome analysis, the mass spectrometer is tuned to detect specific peptide ions related to target proteins. Methods for multiple reactions monitoring and selected reactions monitoring (MRM and SRM, respectively) make it possible to select peptide ions with specified mass-to-charge characteristics at the first and second stages of tandem mass spectrometry. Such methods have several undeniable advantages in comparison with panoramic MS analysis. Bao and colleagues applied MRM with isotopically labeled peptide standards to detect and quantify the staphylococcal enterotoxin SEB in raw chicken [82]. Three peptides were used as a standard, which made it possible to achieve high detection accuracy [83].

The extraction of proteins for LC-MS/MS-based analysis involves using gels or long-term immunoaffinity purification to obtain a pure sample for analysis. Anjelkovich and colleagues presented a combination of LC-MS/MS and in silico, solid-phase extraction via MRM [84], eliminating some shortcomings of the conventional extraction methods. This method was used to identify SEB and SEA in various cultures. The proposed approach allows the detection of toxins in milk samples, while the limits of detection of SEA and SEB were 8 and 4 ng/g, lower than that achieved with many other identification methods (for example, using ultra-high-performance liquid chromatography with tandem mass spectrometry in the article by Muratovic et al. [79]).

The LC-SRM method is considered the most sensitive [85]. Dupre et al. [86] used it for the absolute quantification of eight toxins in food matrices: ricin, ETX, SEA, SEB, SED, *Shigella dysenteriae* shigatoxin, STX1 and STX2 (Escherichia coli), and CDT (*Campylobacter jejuni*). The experimental results confirmed the method’s high sensitivity to toxin concentrations corresponding to food poisoning. In the same work, a unique author’s method for detecting enterotoxins was presented: immuno-LC-MS/MS in combination with PRM. Antitoxin monoclonal antibodies were covalently bound to magnetic elements after which, upon contact with isotope-labeled toxins, selective recognition and binding of the latter occurred. Thus, using the quadrupole–Orbitrap instrument, at least seven peptides were detected for each of the three toxins (ricin, SEB, and Clostridia epsilon toxin). The advantage of this method is its rapidity; in the experiment, which took 5 h, the limit of quantitation was reached at the level of 1 ng ml-1 or lower.

Mass spectrometers are very sensitive quantitative instruments with an attomole detection limit; however, determining the threshold between signal and noise in MS signals is often challenging. Although mass spectrometry cannot provide the depth of coverage of high-throughput sequencing, this method is actively used to identify the proteomes of bacterial strains. Sequencing techniques are inferior to proteomics based on mass spectrometry since proteins can be altered post-translationally, and their structure and function will be altered accordingly. This information cannot be read from gene sequences or transcripts.

The systemic analysis of the literature was performed using an automated text-mining tool and manual approaches. It revealed that proteomic methods have the potential to identify differentially regulated bacterial proteins in food samples or in response to drugs and disinfectants as well as to describe the mechanisms of biofilm formation and the host–parasite relationship. The current technologies offer two major scenarios of food production proteomic profiling. The first one involves the comprehensive, rapid, and robust analysis of key markers of food production status to detect bacterial strains and/or markers of their resistance [87]. The second scenario is to develop chromatographic approaches to perform deep proteomic profiling and overcome the current limitations in the frequency of scans and the complexity of biological samples. Quantitative profiling will allow for the solving of the fundamental problems with high applied potential, for example, tracking in detail the process of biofilm formation by microbial communities in food production conditions, its speed, and the best approaches to limit it [88]. The equipment needed to implement the scenarios described above is complex, expensive, and requires capacities for sample preparation and computing power for data analysis. Therefore, only the cooperation of the food production industries with scientific laboratories will provide the best insights for food safety development. The application of proteomics will be helpful to uplift human health and develop the fields of animal production, agriculture, food processing, and storage [89].

## 4. Conclusions

Based on the above, the proteomics approach serves as a complement to the genomic and transcriptomic approaches. Proteomics is effective in the study of complex mapping of bacterial proteomes, post-translational modifications of proteins, and pathogen–host interactions, as well as in antimicrobial resistance and the discovery of new protein biomarkers. A knowledge base is needed in which bacterial genes are annotated in the context of antibiotic resistance. Such an information resource will allow the creation of more sensitive methods for assessing the state of food production and considering the variety of bacteria and the resistance of specific strains to disinfectants. Proteomic bacterial resistance research is at its early stages but shows great perspectives. However, the most advantageous strategy is the use of a multi-omics approach. It provides cross-validation of the results and gives a complete understanding of the molecular processes characterizing the food production bacterial communities. The multi-omics approach can undercover the mechanisms of biofilm resistance, tracking the most dangerous combinations of bacterial strains and surfaces on which they are attached.

The text-mining approach gives us an opportunity to process large amounts of papers and to extract the most valuable information about the proteomic basics of bacterial resistance. The semantic networks are useful instruments for scientific literature surfing.

## Figures and Tables

**Table 1:** Accumulated information on the genes and proteins associated with resistance development in three types of microorganisms.

Organism	Marker (Gene/Protein)	UniProt ID	Resistance	Reference
*Salmonella enterica*	tetA, tetG and tetC, TetR	A0A1Z1VWZ4 *, A3RLS9 *, A0A3G1TUX2 *, A0A1Z1VX01 *	Tetracycline	[51,52]
	gyrA	A0A447PDY4 *	Nalidixic acid	[51]
	mcr-1	A0A1P8DNG0 *	Colistin	[53]
	bla(PSE) and bla(TEM)	K9M2D2 *, A0A7D7QQ45 *	Ampicillin, penicillin antibiotics	[52]
	cat1, cat2 and floR	Q3HNN0, Q7BD42, A0A3K0TB41 *	Chloramphenicol	[52]
	strA, strB and aadA	A0A5C2D198 *, C4NVB8 *, Q02865	Streptomycin	[52]
	sul1 and sul2	A0A0F6NWV0 *, A0A1S6KR61 *	Sulfizoxazole	[52]
	AcrAB/TolC, including acetylated lysine (PTM)	A0A759DN94 *	Fluoroquinolone	[54]
			Quaternary ammonium compounds, chloramphenicol, tetracycline, ampicillin	[55,56]
	AcrB	A0A379QMS3 *	Ciprofloxacin	[57]
	FabI	A0A5Z3DTY7 *	Triclosan	[56]
	TolB, ElaB, TolC, GrxB, Tpx, Tsx, AhpF, AhpC, NfnB	A0A447MVS8 *, E8XEF3 *, Q54001, A0A6C8EYP6 *, A0A3F3I872 *, A0A379QQ98 *, A0A8E9PK94 *, A0A379WCI5 *, A0A447MWB6 *	Wide range of antimicrobial agents	[56]
	AcnA	A0A711ME57 *	Quaternary ammonium compounds	[56]
	RpoE, CpxR	D0ZSY9 *, A0A5Z8M962 *	Chlorhexidine	[58]
	csgD, bcsA, ardA	O54294, A0A2T8TBA6 *, A0A410J986 *	Quaternary ammonium compounds	[31]
*Listeria monocytogenes*	FosX	Q8Y6I2	Fosfomycin	[59]
	FosE	A0A3T2HNE9 *	Tetronazine	
	FosI	A0A5M3ENG4 *	Bleomycin	
	LiaR	A0A0E1R5S4 *	Nisin	[60]
	TetM, TetS	Q5WMA8, Q48791	Tetracyclines, tellurite	[61]
	dfrD, dfrG, dhfr	Q79CE5	Trimethoprim	[62]
	ErmA(TR), ErmB, ErmC	K4NRN0 *, K4NU76 *	Erythromycin, quaternary ammonium compounds	[63,64]
	lnuB	A0A4D6D220 *	Lincosamide	[65]
	PBP1	Q8Y614	Cephalosporins	[66,67]
	LysR, LytR, LytTR, Rgg	A0A5M2ZBL1 *, A0A6W3T4B1 *, A0A5Y7CVQ1 *, A0A3H2VUB6 *	Cationic antimicrobial peptides (CAMP)	[68]
	MerR	A0A3T2B4E2 *	Mercury resistance	[61]
	bcrA, bcrB, bcrC, qacH, qacA	A0A5Y1L6S6 *, I6ZWK8 *, I7B1C4 *, T2KSX9 *, T1YPL4 *	Quaternary ammonium compounds	[69]
*Pseudomonas aeruginosa*	gyrB	P13364	Aminocoumarin	[70]
	aadA, rpsL	A0A844NVA2 *, Q9HWD0	Aminoglycosides	[71,72]
	blaCTX–M, PBP1a, PBP1b, oprD	A0A0M4CJ048, Q07806, A0A165VXD8 *, P32722	Beta lactams	[73]
	gyrA, gyrB, patA, patB, parC, parE, emrA, emrB, mdtK, mfd	P48372, P13364, A0A5E7MVR1 *, Q5BU34, Q9HUK1, Q9HUJ8, A0A5E7FSX8 *, A0A5M9IUB7 *, A0A0B7DI98 *, Q9HZK3	Fluoroquinolones	[73,74]
	mdtD, mdtG, mdtH, glpT, murA	A0A5E6SQR7 *, A0A5E6YH70 *, A0A5E6QA20 *, Q9HTV5, Q9Z3Z6	Fosfomycin	[73]
	vanA	O05616	Glycopeptide antibiotics	[73]
	arnA, bacA, bcrA, liaR, mprF, phoP, phoQ, pmrA, pmrB, pmrE, pmrF, rosB, floR, lpxA, lpxC, cls, pgsA, rpoC	Q02R25, Q02LA5, Q937U9, A0A8D9KX77 *, A0A1B5E8X0 *, Q9I4F9, Q9I4F8, Q9HV32, Q9HV31, A0A0C6ED02 *, A0A519EUM6 *, A0A485GZQ4 *, A0A4D6RIK8 *, Q9X6P4, P47205, A0A2K9MAE4 *, P45419, P19176	Lipopeptides	[73,75,76],
	carA, macA, macB	Q88DU5, A0A5E7QUJ5 *, Q88F88	Macrolides	[73]
	mefA	A0A383RT01 *	Macrolide-Lincosamide-Streptogramin B	[73]
	catA1, catB3, catI, floR	T2HGZ6 *, V5LZV8 *, Q8VPF3, A0A4D6RIK8 *	Fenicol	[73]
	rpoB	P19175	Rifampin	[73]
	sul1	A0A2R4S1K0 *	Sulfonamides	[73]
	tetA, tetB(P), tetG, tetM	A0A4P2USE6 *, A0A4Y5T9V5 *, Q9X685, A0MZ57 *	Tetracycline	[73,77]
	katG, kasA	Q88GQ0, A0A379J0D7 *	Isoniazid	[73]
	triA, triB, triC, opmH	P72156, A0A8F9V618 *, A0A8G1K8C1 *, A0A8G6KPN1 *	Triclosan	[73,78]

*—unreviewed proteins according to the UniProt database.

## Data Availability

Not applicable.
